# Determination of *N*-Acetyl-l-cysteine Ethyl Ester (NACET) by Flow Injection Analysis and Spectrophotometric Detection Using Different Thiol-Sensitive Ligands

**DOI:** 10.3390/molecules26226826

**Published:** 2021-11-11

**Authors:** Lea Kukoc-Modun, Tomislav Kraljević, Dimitrios Tsikas, Njegomir Radić, Darko Modun

**Affiliations:** 1 Department of Analytical Chemistry, Faculty of Chemistry and Technology, University of Split, Ruđera Boškovića 35, 21000 Split, Croatia; njego.rad.st@gmail.com; 2 Department of Chemistry, Faculty of Science and Education, University of Mostar, Matice hrvatske bb, 88000 Mostar, Bosnia and Herzegovina; kralj.tomo.mo@gmail.com; 3 Institute of Toxicology, Core Unit Proteomics, Hannover Medical School, Carl-Neuberg-Str. 1, 30625 Hannover, Germany; tsikas.dimitros@mh-hannover.de; 4 Department of Pharmacy, School of Medicine, University of Split, Šoltanska 2, 21000 Split, Croatia; Drmodun@gmail.com

**Keywords:** *N*-acetyl-l-cysteine ethyl ester, neocuproine, bicinchoninic acid, bathocuproine disulfonic acid, flow injection analysis, spectrophotometric determination

## Abstract

A new flow injection spectrophotometric method for the determination of *N*-acetyl-l-cysteine ethyl ester (NACET) was developed and validated. The method is based on the reduction of Cu(II)-ligand complexes to chromophoric Cu(I)-ligand complexes with the analyte. The studied ligands were neocuproine (NCN), bicinchoninic acid (BCA) and bathocuproine disulfonic acid (BCS). The absorbance of the Cu(I)-ligand complex was measured at 458, 562 and 483 nm for the reactions of NACET with NCN, BCA and BCS, respectively. The method was validated in terms of linear dynamic range, limit of detection and quantitation, accuracy, selectivity, and precision. Experimental conditions were optimized by a univariate method, yielding linear calibration curves in a concentration range from 2.0 × 10^−6^ mol L^−1^ to 2.0 × 10^−4^ mol L^−1^ using NCN; 2.0 × 10^−6^ mol L^−1^ to 1.0 × 10^−4^ mol L^−1^ using BCA and 6.0 × 10^−7^ mol L^−1^ to 1.2 × 10^−4^ mol L^−1^ using BCS. The achieved analytical frequency was 90 h^−1^ for all three ligands. The method was successfully employed for NACET determination in pharmaceutical preparations, indicating that this FIA method fulfilled all the essential demands for the determination of NACET in quality control laboratories, as it combined low instrument and reagent costs with a high sampling rate.

## 1. Introduction

*N*-Acetylcysteine ethyl ester (NACET) is a lipophilic, charge-free, cell-permeable cysteine derivative with highly improved pharmacological properties in comparison with its congener *N*-acetyl-l-cysteine (NAC). At physiological pH values, the free carboxylic group of NAC is almost entirely negatively charged. Thus, active transmembrane transport is required, which is clearly limited. The esterification of the carboxyl group of NAC increases the lipophilicity of NAC and improves its pharmacokinetics. NACET has the potential to substitute NAC as a mucolytic agent but also as an antioxidant, a supplier of glutathione (GSH) and a paracetamol antidote [1,2].

A recent report showed that NAC did not act as a GSH enhancer in human endothelial cells in a dose-dependent manner. Instead, the treatment with NACET had an evident ceiling effect. The levels reached by intracellular GSH after treatments with NACET resulted in a balance between the induction of its synthesis (by increased cysteine) and enzyme inhibition (by increased intracellular NAC) [3]. Therefore, it is possible that high doses of NACET could have a paradoxical effect by decreasing rather than increasing GSH. This finding highlights the importance of determining NACET levels in both biological samples and pharmaceutical formulations.

NACET and its metabolites can be analyzed in biological samples by high-performance liquid chromatography (HPLC) after derivatization [1]. In pharmaceutical preparations NACET can be analyzed by kinetic spectrophotometry, generating chromogenic copper(I)Ln complexes with different ligands (Ln): neocuproine (NCN), bicinchoninic acid (BCA) or bathocuproine disulfonic acid (BCS) [4]. The reported kinetic methods are based on the reduction of the Cu(II)-ligand complex to Cu(I)-ligand complex by NACET. The redox reactions are fast, proceed in the broad pH range, and provide stable signals. Therefore, the kinetics of those reactions between the Cu(II)-ligand and NACET suggest that the development of a new, flow injection analysis (FIA) method for the detection of NACET would be feasible [4].

FIA became a versatile instrumental tool for the quality control of pharmaceuticals in the 21st century due to its instrumentation simplicity, inexpensive determination and high throughput capacities [5]. Pre-existing batch instruments can be easily converted to flow through by either home-made or commercially available cells [6]. Nevertheless, FIA enables the development of cleaner analytical methods to provide greener analytical chemistry [7]. The development of flow-based techniques has brought a new dimension to analytical chemistry, allowing the measurements to be carried out faster and with minimum intervention from the analyst, as well as minimum reagent(s) consumption and lower waste generation. In order to fulfill the demands of green chemistry analysis, recently, some improved concepts of flow-based analytical systems were proposed: multi-commutation in flow analysis [8], multisyringe flow injection analysis [9], all injection analysis [10], multi-pumping in flow analysis [11], sequential injection chromatography [12], sequential injection lab at valve [13], stopped-in-loop flow analysis (SILFA) [14], stopped-in-dual-loop flow analysis (SIDLFA) [15], simultaneous injection effective mixing flow analysis (SIEMA) [16,17] and programmable flow injection (pFI) [18].

As NACET is a relatively new and experimental compound, there are currently no registered products intended for human consumption available on the market. Therefore, there is a very limited number of published methods for the detection of NACET, either as a pure compound or in a pharmaceutical sample. The aim of the present study is the development and validation of a new FIA method for the determination of NACET with a visible spectrophotometric detection with the use of Cu(II) and different ligands: NCN, BCA and BCS.

## 2. Results and Discussion

The reactions involved in the present study are based on the one-step redox reaction (Equations (1)–(3)), in which NACET (RSH compound) reduces the Cu(II)-ligand complex to the Cu(I)-ligand complex, which exhibits absorption maxima at 458 nm [19], 562 nm [20] and 483 nm [21] for NCN, BCA and BCS, respectively:

(1) $$2\mathrm{RSH}+2{\left[\mathrm{Cu}{\left(\mathrm{NCN}\right)}_{2}\right]}^{2+}⇄\;\mathrm{RSSR}+2{\left[\mathrm{Cu}{\left(\mathrm{NCN}\right)}_{2}\right]}^{+}+{2H}^{+}$$

(2) $$2\mathrm{RSH}+2{\left[\mathrm{Cu}{\left(\mathrm{BCA}\right)}_{2}\right]}^{2-}⇄\;\mathrm{RSSR}+2{\left[\mathrm{Cu}{\left(\mathrm{BCA}\right)}_{2}\right]}^{3-}+{2H}^{+}$$

(3) $$2\mathrm{RSH}+2{\left[\mathrm{Cu}{\left(\mathrm{BCS}\right)}_{2}\right]}^{2-}⇄\;\mathrm{RSSR}+2{\left[\mathrm{Cu}{\left(\mathrm{BCS}\right)}_{2}\right]}^{3-}+{2H}^{+}$$

### 2.1. Optimization of the Chemical Conditions

#### 2.1.1. Neocuproine (NCN) Ligand

The effect of pH on peak height was examined in the pH range 2.0–8.0, using a Britton–Robinson buffer solution. An optimum pH of 5.0 was chosen for further measurements. The effect of the temperature on the reaction was studied in the range 20–40 °C. The reaction was found to be practically independent of temperature in the examined temperature range, and the analyses were performed at room temperature (25 °C). The optimal molar ratio between Cu(II) and NCN was 1/2.4, resulting in final concentrations of *c*(Cu^2+^) = 6.0 × 10^−4^ mol L^−1^ and *c*(NCN) = 1.44 × 10^−3^ mol L^−1^ (Table 1).

#### 2.1.2. Bicinchoninic Acid (BCA) Ligand

The effect of pH on peak height was examined in the pH range 2.0–8.0. An optimum pH of 7.0, using a phosphate-buffered solution, was chosen for further measurements. The reaction was found to be temperature-dependent, but stable at room temperature (25 °C). The optimal molar ratio between Cu(II) and BCA was 1/2, resulting in final concentrations of *c*(Cu^2+^) = 4.0 × 10^−4^ mol L^−1^ and *c*(BCA) = 8.0 × 10^−4^ mol L^−1^ (Table 1).

#### 2.1.3. Bathocuproine Disulfonic Acid (BCS) Ligand

The effect of pH on peak height was examined in the pH range 2.0–8.0. An optimum pH of 5.0, using an acetate buffer solution, was chosen for further measurements. The reaction was found to be practically independent of temperature in the examined temperature range, and the analyses were performed at room temperature (25 °C). The optimal molar ratio between Cu(II) and BCS was 1/2, resulting in final concentrations of *c*(Cu^2+^) = 4.0 × 10^−4^ mol L^−1^, *c*(BCS) = 8.0 × 10^−4^ mol L^−1^ (Table 1).

### 2.2. Optimization of the FIA Conditions

The values of the main FIA parameters (carrier and reagent flow rates, reaction coil length, reagent concentration and sample volume) represent the best compromise between absorbance signal, repeatability and sample throughput (Table 1). The effects of carrier stream flow, reagent stream flow, injection sample volume and reaction coil length on peak heights are shown in Supplementary Materials Figure S1.

#### 2.2.1. Neocuproine (NCN) Ligand

The carrier and reagent flow rate were studied by varying the rotation speed of the peristaltic pump. The optimal values of 6.0 mL min^−1^ and 2.0 mL min^−1^ for the carrier and reagent flow rate, respectively, were chosen for further experiments. The injected sample volume into the carrier stream showed an optimum value of 500 µL due to the maximum absorbance signal and better run-to-run reproducibility. The overlap of the reagent and analyte zone occurred in the reaction coil where the proposed redox reaction took place. A reaction coil length of 40 cm was sufficient for the redox reaction to almost be completed.

#### 2.2.2. Bicinchoninic Acid (BCA) Ligand

The optimal values of 5.0 mL min^−1^ and 1.5 mL min^−1^ for the carrier and reagent flow rate, respectively, were chosen for further experiments. The injected sample volume into the carrier stream showed an optimum value of 500 µL due to the maximum absorbance signal and better run-to-run reproducibility. A reaction coil length of 40 cm was sufficient for the redox reaction to come near to completion, with minimal dispersion of the signal.

#### 2.2.3. Bathocuproine Disulfonic Acid (BCS) Ligand

The optimal values of 5.0 mL min^−1^ and 1.5 mL min^−1^ for carrier and reagent flow rate, respectively, were chosen for further experiments. The injected sample volume into the carrier stream showed an optimum value of 500 µL due to the maximum absorbance signal and better run-to-run reproducibility. A reaction coil length of 30 cm was sufficient for the redox reaction to almost be completed.

### 2.3. Analytical Characteristics

**Figure 1:**
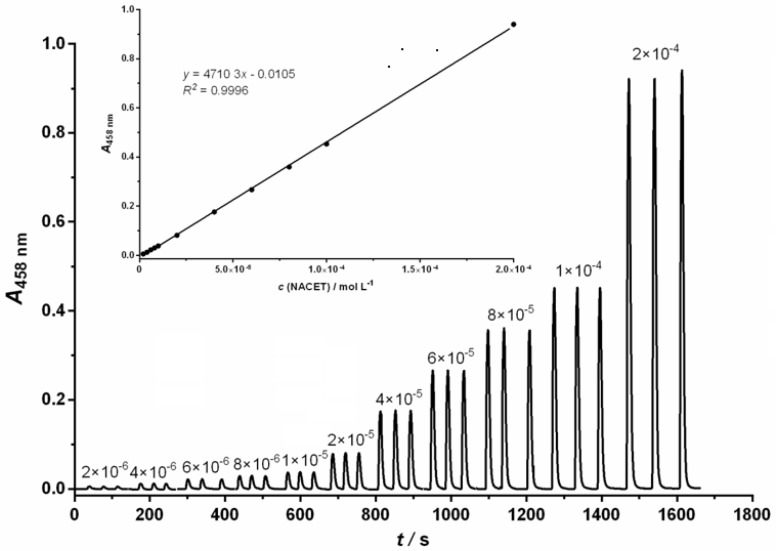
Diagram chart and calibration curve for spectrophotometric determination of NACET in the concentration range of 2.0 × 10^−6^ mol L^−1^ to 2.0 × 10^−4^ mol L^−1^. Experimental conditions: *c*(Cu^2+^) = 6.0 × 10^−4^ mol L^−1^; *c*(NCN) = 1.44 × 10^−3^ mol L^−1^; pH = 5.0; *t* = 25 °C, carrier stream flow = 6.0 mL min^−1^; reagent stream flow = 2.0 mL min^−1^.

Under the optimized conditions, the proposed FIA method showed a good linear response in the range from 2.0 × 10^−6^ mol L^−1^ to 2.0 × 10^−4^ mol L^−1^ of NACET for NCN; 2.0 × 10^−6^ mol L^−1^ to 1.0 × 10^−4^ mol L^−1^ of NACET for BCA and 6.0 × 10^−7^ mol L^−1^ to 1.2 × 10^−4^ mol L^−1^ of NACET for BCS. Figure 1 shows a representative example of an FIA gram chart and calibration curve (inlet) for the detection of NACET using the NCN ligand.

The limits of detection (LOD), regression equations with corresponding coefficients, relative standards deviation and analytical frequency are shown in Table 2.

### 2.4. Interference Studies

The selectivity was examined under the optimized conditions (Table 1) by analyzing samples of the NACET test concentration of 4.0 × 10^−5^ mol L^−1^ containing some substances commonly used in pharmaceutical formulations. The tolerance limit was taken as the molar ratio of NACET to the foreign substance which causes an error of ± 5% in recorded peak height. The selectivity results for each ligand are summarized in Table 3.

### 2.5. Recovery Studies

As there are no NACET-containing pharmaceutical formulations available on the market, recovery studies were evaluated in placebo formulations by adding NACET in quadruplicate at four concentrations (Table 4).

The recovery (accuracy) of the developed FIA method for the determination of NACET was in the range from 98.8% to 102.6% with RSD < 2% for all three ligands. The results supported the accuracy and precision of the developed method, as well the absence of the significant interference from excipients in the used samples. 

### 2.6. Comparison between the Ligands

We successfully developed and validated a new, simple and rapid FIA method for the spectrophotometric determination of NACET with the use of Cu(II) and different ligands: NCN, BCA and BCS. While our results suggest that all three ligands are almost equivalent with respect to sensitivity, precision and speed, there are some differences between these ligands that must be addressed. The higher sensitivity of BCS, indicated by the lowest LOD and LOQ, is likely to be due to the higher electron delocalization of the aromatic system in BCS, as we suggested previously [4]. Both BCS and NCN are temperature-independent, and the redox potential of Cu(II)/Cu(I) in the solution of NCN or BCS indicates the selectivity of the method, as only the reducing substances with a formal (standard) potential lower that 0.6 V have the thermodynamic predisposition to interfere with the proposed method. However, both NCN and BCA could be susceptible to interference by some substances commonly used in pharmaceutical formulations, such as lactose, sucrose, citric acid, or tartaric acid (Table 3). Notably, our recovery studies showed an acceptable level of accuracy in our tested samples for all three ligands. The price of NCN is only 20% that of BCA and BCS, but the superior selectivity of BCS could suggest this ligand as a first choice for adapting this FIA method for the determination of NACET in pharmaceutical formulations, as the consumption of the ligand is fairly small.

## 3. Materials and Methods

### 3.1. Reagents and Solutions

All reagents used throughout this study were of analytical grade, and all solutions were prepared with water from a Millipore Milli-Q system (Saint Quentin, Yvelines, France).

The synthesis, purification, mass spectrometry, ^1^HNMR, infrared spectrometry and polarimetry characterization of NACET (C_7_H_13_NO_3_S, MW 191.2, mp 44.1–44.5 °C) were reported previously [22].

The stock solution of *N*-acetylcysteine ethyl ester (*c* = 1.0 × 10^−2^ mol L^−1^) was prepared by dissolving 0.1912 g of pure substance in Britton–Robinson buffer solution (pH = 2) and diluting to 100.0 mL volume. The prepared stock solution was stored at 4 °C, and working standard solutions of lower concentrations were prepared daily by diluting the stock solution with Britton–Robinson buffer solution (pH = 2).

The Britton–Robinson buffer solution (pH = 2, *c* = 4.0 × 10^−2^ mol L^−1^) was prepared by mixing appropriate volumes of acetic, boric and phosphoric acid. The buffer solutions with other required pH values were adjusted by adding a 2.0 mol L^−1^ sodium hydroxide solution to the prepared Britton–Robinson buffer solution. 

The phosphate-buffered solutions (pH between 6.0 and 8.0) were prepared by mixing 5.0 × 10^−2^ mol L^−1^ solution of potassium hydrogen phosphate with 5.0 × 10^−2^ mol L^−1^ solution of potassium dihydrogen phosphate in appropriate proportions.

Acetate buffer solutions (pH between 3.5 and 6.0) were prepared by mixing appropriate volumes of 5.0 × 10^−2^ mol L^−1^ sodium acetate solution and 5.0 × 10^−2^ mol L^−1^ acetic acid. The final buffer solutions of exact pH were adjusted by addition of 2.0 mol L^−1^ sodium hydroxide solution to prepared buffer solutions.

A stock solution 1.0 × 10^−2^ mol L^−1^ of Cu(II) was prepared by dissolving 0.1248 g of copper sulphate pentahydrate in water and diluting to the mark in a 50.0 mL standard flask.

Neocuproine is slightly soluble in water, but complexation with the Cu(II) improves its solubility [19]. Therefore, after optimization of the molar ratio of neocuproine and Cu(II) (Table 1), the copper(II)-neocuproine reagent was prepared by dissolving 25.0 mg of copper sulphate pentahydrate and 50.0 mg of neocuproine in 20 mL of Britton–Robinson buffer solution (pH = 5) to the final volume of 100.0 mL.

Bicinchoninic acid solution (BCA), 4.0 × 10^−3^ mol L^−1^ was prepared by dissolving 77.6 mg of bicinchoninic acid disodium salt in phosphate-buffered solution (pH = 7) in 50 mL standard flask. According to the optimization of chemical parameters, BCA reagent solution consisted of 4.0 × 10^−4^ mol L^−1^ Cu(II) and 8.0 × 10^−4^ mol L^−1^ BCA in phosphate-buffered solution (pH = 7).

Bathocuproine disulfonic acid (BCS) solution was prepared by dissolving 58.2 mg of disodium salt up to a 50.0 mL volume with deionized water. BCS reagent solution was prepared in acetate buffer solution (pH = 5) with a final concentration of components: 4.0 × 10^−4^ mol L^−1^ Cu(II) and 8.0 × 10^−4^ mol L^−1^ BCS.

Placebo tablets containing starch, lactose, magnesium stearate and Povidon K-30 (Pliva Hrvatska Ltd., Zagreb, Croatia) were used for recovery studies. Ten tablets were weighed and pulverized. A portion of the powder, equal to the weight of the average tablet, was dissolved in 300 mL of water, filtered through filter paper and the filtrate collected in a 500-mL volumetric flask and diluted with water.

### 3.2. Apparatus

A double beam UV-Vis spectrophotometeric detector (UV-1601 Shimatzu Kyoto, Japan) equipped with a flow-through cell (10 mm optical path) with internal volume of 160 μL was used for all absorbance measurements. The output signals were recorded by coupling the spectrophotometer to a computer equipped with Hyper UV-Vis software provided by Shimadzu. The data acquisition frequency was 1 Hz. The absorbance signal was measured as a peak height. Three replicates per sample or standard were made in all instances, unless stated otherwise.

The pH adjustments and measurements were made with a Mettler Toledo SevenMulti potentiometer (Mettler Toledo, Schwerzenbach, Switzerland) fitted with a combined glass electrode Mettler Toledo InLab^®^413. The temperature optimization was carried out in a temperature controllable water bath accurate to ± 0.5 °C.

### 3.3. FIA Manifold and Procedure

**Figure 2:**
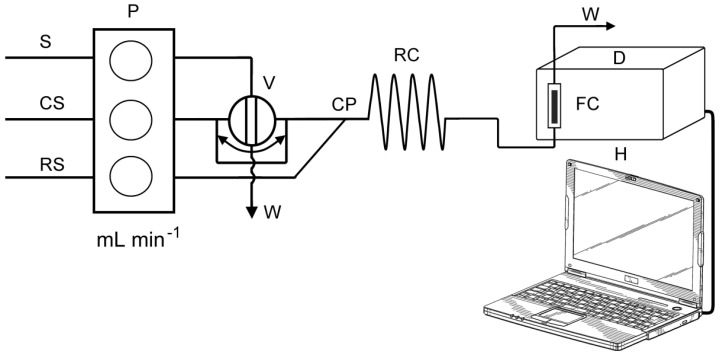
Schematic diagram of the flow injection manifold: S—standard solution, CS—carrier (ultra pure H_2_O), RS—reagent (reagents depends on the ligand), P—peristaltic pump, V—injector valve (loop = 500 µL), CP—confluence point, RC—reaction coil (i.d. = 0.8 mm), D—spectrophotometric detector equipped with flow cell (FC), (VFC = 160 µL), H—computer, W—waste.

A schematic diagram of the FIA manifold used in the present work is shown in Figure 2. The FIA manifold was previously described in more detail [23]. The Ismatec IPC eight-channel peristaltic pump (Ismatec, Zurich, Switzerland) was used to pump the sample, carrier and reagent solutions. A rotary valve (Rheodyne, Model 5020, Anachem, Luton, UK) was used for injecting standards and samples into the carrier stream. The manifold was built up with polytetrafluoroethylene (PTFE) tubes with 0.8 mm bore. For the optimization part of the experiment, the manifold was mainly thermostated (reagent, carrier and sample containers, reaction coil). It was found that, for NCN and BCS ligands, the reactions were temperature-independent, and for BCA ligand, the reaction was stable at room temperature (25 °C). Therefore, at further measurements the manifold was not thermostated, and all measurements were performed at room temperature (25 °C).

A carrier solution (CS), deionized water, was pumped into analytical line at flow rate of 5.0 mL min^−1^ (for BCA and BCS ligands) or 6.0 mL min^−1^ (for NCN ligand). Reagent solution (RS) was pumped at rate of 1.5 mL min^−1^ (for BCA and BCS ligands) or 2.0 mL min^−1^ (for NCN ligand). An aliquot of sample solution (500 μL) was introduced into a carrier stream by loop–valve injector, and thus formed sample zone merged into a reagent stream at confluence point (CP). Subsequently, the redox reaction took place as the final stream flowed into the reaction coil (RC) (i.d. 0.8 mm, length: 40 cm for BCA and NCN ligands or 30 cm for BCS ligand) corresponding to a volume of 200 μL and 150 μL, respectively. The formed colored complex reached the flow cell unit positioned in the optical path of the UV/Vis spectrophotometer. The absorption of the formed complex was continuously recorded at 458 nm (for NCN ligand), 562 nm (BCA) and 483 nm (BCS).

## 4. Conclusions

The reported FIA spectrophotometric method for the determination of *N*-acetyl-l-cysteine ethyl ester (NACET) is sensitive, accurate, precise and rapid. In this method, NACET reduces Cu(II)-ligand complexes to chromophoric Cu(I)-ligand complexes. All three studied ligands, neocuproine (NCN), bicinchoninic acid (BCA) and bathocuproine disulfonic acid (BCS), showed an adequate sensitivity, precision and accuracy. The method was successfully employed for NACET determination in pharmaceutical preparations, indicating that this FIA method fulfils all the essential demands for the determination of NACET in quality control laboratories. Furthermore, the reported method offers low instrument and reagent costs combined with a high sampling rate.

## Figures and Tables

**Table 1:** Optimization of chemical parameters and manifold conditions for the proposed FIA method.

Variable	Studied Range	Optimum Conditions		
		NCN	BCA	BCS
Wavelength (nm)	400–800	458	562	483
pH	2.0–8.0	5.0	7.0	5.0
Temperature (°C)	20–40	25	25	25
Molar ratio Cu(II)/ligand	1/1.0–1/3.5	1/2.4	1/2	1/2
CS ^1^ flow rate (mL min^−1^)	0.5–6.0	6.0	5.0	5.0
RS ^2^ flow rate (mL min^−1^)	1.0–4.0	2.0	1.0	1.0
Injection sample volume (µL)	100–1000	500	500	500
Reaction coil length (cm)	30–525	40	40	30

^1^ Carrier stream. ^2^ Reagent stream.

**Table 2:** Analytical characteristics of the developed FIA method.

	NCN	BCA	BCS
Linear range (mol L^−1^)	2.0 × 10^−6^ −2.0 × 10^−4^	2.0 × 10^−6^ −1.0 × 10^−4^	6.0 × 10^−7^ −1.2 × 10^−4^
Regression equation	*y* = 4710*x* −0.0105	*y* = 3850*x* −0.0108	*y* = 8437*x* −0.003
LOD (mol L^−1^) ^1^	6.0 × 10^−7^	8.3 × 10^−7^	2.2 × 10^−7^
Correlation coefficient, *R*^2^	0.9996	0.9993	0.9995
Relative standard deviation ^2^, RSD (%)	0.41	0.70	0.36
Analytical frequency (h^−1^)	90	90	90

^1^ LOD was calculated as three standard deviations of a blank divided by the slope of the calibration curve. ^2^ RSD of measured peak heights for 20 injections containing 4.0 × 10^−5^ mol L^−1^ of NACET.

**Table 3:** The effect of possible interfering substances for the developed method.

Substance	Optimum Conditions ^1^		
	NCN	BCA	BCS
Glucose	1:500	1:500	1:500
Fructose	1:500	1:500	1:500
KNO_3_	1:500	1:500	1:500
Lactose	1:5	1:500	1:500
Sucrose	1:5	1:50	1:100
Citric acid	1:10	1:1	1:250
Tartaric acid	1:50	1:5	1:500
Na_2_SO_4_	1:500	1:500	1:500
Na-citrate	1:500	1:1	1:250
H_3_BO_3_	1:500	1:500	1:100

^1^ Molar ratio of NACET to foreign substance.

**Table 4:** Testing the accuracy of the new method for the determination of NACET.

		NCN		BCA		BCS	
Sample	Added (µg mL^−1^)	Found (µg mL^−1^)	Recovery (%)	Found (µg mL^−1^)	Recovery (%)	Found (µg mL^−1^)	Recovery (%)
Placebo	0	0	-	0	-	0	-
	50	50.6 ± 0.7	101.2	49.4 ± 0.5	98.8	50.3 ± 0.6	100.6
	100	102.6 ± 1.2	102.6	101.1 ± 1.0	101.1	102.3 ± 1.4	102.3
	150	153.8 ± 2.0	102.5	148.9 ± 2.2	99.3	152.9 ± 2.5	101.9
	200	204.1 ± 2.4	102.1	203.4 ± 3.1	101.7	203.9 ± 3.0	102.0

## Data Availability

The data presented in this study are available on request from the corresponding author.

## Supplementary Materials

The following are available online. Figure S1: Effects of carrier stream, CS, flow (panel A), reagent stream, RS, flow (panel B), injection sample volume (panel C) and reaction coil length (panel D) on peak heights of 4 × 10^−5^ mol L^−1^ of NACET using neocuproine (NCN), bicinchoninic acid (BCA) or bathocuproine disulfonic acid (BCS) as the ligand.

## Sample Availability

Samples of the compound NACET is available from the author (Dimitrios Tsikas).
