# Supplementary material for: Determination of N-Acetyl-l-cysteine Ethyl Ester (NACET) by Flow Injection Analysis and Spectrophotometric Detection Using Different Thiol-Sensitive Ligands
Source: Molecules. 2021 Nov 11;26(22):6826. doi: 10.3390/molecules26226826 (PMC8624116; doi:10.3390/molecules26226826)

Figure S1.

Effects of carrier stream, CS, flow (panel A), reagent stream, RS, flow (panel B), injection sample volume (panel C) and reaction coil length (panel D) on peak heights of 4×10^{-5} mol L^{-1} of NACET using neocuproine (NCN), bicinchoninic acid (BCA) or bathocuproine disulfonic acid (BCS) as the ligand. Absorbance was measured at 458 nm, 562 nm or 483 nm for NCN, BCA or BCS, respectively.

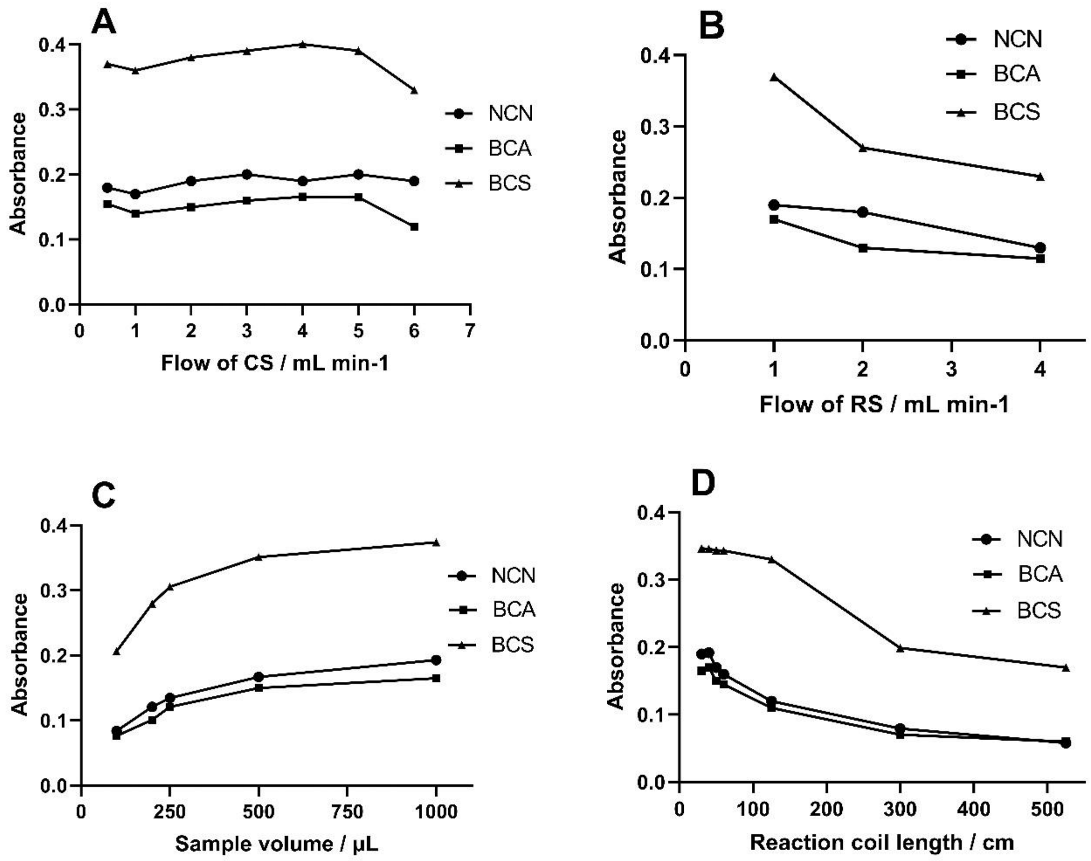

Supplement: Supplementary file 1 [file molecules-26-06826-s001.zip › molecules-1427063-supplementary.pdf]
